# Heme arginate improves reperfusion patterns after ischemia: a randomized, placebo-controlled trial in healthy male subjects

**DOI:** 10.1186/1532-429X-14-55

**Published:** 2012-08-02

**Authors:** Martin Andreas, Albrecht Ingo Schmid, Daniel Doberer, Kiril Schewzow, Stefan Weisshaar, Georg Heinze, Martin Bilban, Ewald Moser, Michael Wolzt

**Affiliations:** 1 Department of Clinical Pharmacology, Medical University of Vienna, Vienna, Austria; 2 Department of Cardiac Surgery, Medical University of Vienna, Vienna, Austria; 3 MR Center of Excellence, Center for Biomedical Engineering and Physics, Medical University of Vienna, Vienna, Austria; 4 Center for Medical Statistics, Informatics and Intelligent Systems, Medical University of Vienna, Vienna, Austria; 5 Department of Laboratory Medicine, Medical University of Vienna, Vienna, Austria

## Abstract

**Background:**

Heme arginate can induce heme oxygenase-1 to protect tissue against ischemia-reperfusion injury. Blood oxygen level dependent (BOLD) functional magnetic resonance imaging measures changes in tissue oxygenation with a high spatial and temporal resolution. BOLD imaging was applied to test the effect of heme arginate on experimental ischemia reperfusion injury in the calf muscles.

**Methods:**

A two period, controlled, observer blinded, crossover trial was performed in 12 healthy male subjects. Heme arginate (1 mg/kg body weight) or placebo were infused 24 h prior to a 20 min leg ischemia induced by a thigh cuff. 3 Tesla BOLD-imaging of the calf was performed and signal time courses from soleus, gastrocnemius and tibialis anterior muscle were available from 11 participants for technical reasons.

**Results:**

Peak reactive hyperemia signal of the musculature was significantly increased and occurred earlier after heme arginate compared to placebo (106.2±0.6% at 175±16s vs. 104.5±0.6% at 221±19s; p = 0.025 for peak reperfusion and p = 0.012 for time to peak).

**Conclusions:**

A single high dose of heme arginate improves reperfusion patterns during ischemia reperfusion injury in humans. BOLD sensitive, functional MRI is applicable for the assessment of experimental ischemia reperfusion injury in skeletal muscle.

**Trial registration:**

ClinicalTrials: NCT01461512

EudraCT: 2008-006967-35

## Background

Rapid reperfusion is essential for ischemic muscle. However, reperfusion itself can result in additional damage to ischemic tissue [1,2]. One of the key mechanisms is the rise of free oxygen radicals, thereby exceeding the cellular antioxidant capacity and damaging cellular proteins and membranes [3]. In a second step, neutrophil activation and tissue invasion is part of the deleterious cascade of ischemia-reperfusion injury (IRI) [4].

Several strategies to alleviate IRI have been tested in various experimental settings, including mechanical preconditioning and postconditioning, pharmacologic preconditioning, administration of antioxidant substances and treatment with endothelin receptor antagonists [5-10]. As a new therapeutic approach in humans, the induction of the enzyme heme oxygenase 1 (HO-1) may be employed to mitigate IRI [10,11]. HO-1 is the rate-limiting enzyme for the degradation of heme b [12]. Thereby, it produces biliverdin, carbon monoxide (CO) and iron. Recently, the role of HO-1 as a protective enzyme was proposed due to its anti-inflammatory, antioxidant, anti-apoptotic and antiproliferative actions [11,13]. HO-1 is expressed in several organs including endothelial and smooth muscle cells in response to cellular stress conditions [14-16].

IRI may be attenuated by pharmacological HO-1 induction with heme arginate (HA) as shown in a rodent hemorrhagic shock model [11,17-19]. Previous data have confirmed dose-dependent induction of HO-1 mRNA and protein by HA in venous blood of healthy subjects [20,21].

In this therapeutic exploratory study, we aimed to evaluate the effects of HA on skeletal muscle IRI in healthy humans. Blood oxygen level dependent (BOLD) functional magnetic resonance imaging (fMRI) was used to measure alterations in tissue oxygenation with a high spatial and temporal resolution [22,23]. fMRI has previously been used to assess perfusion of the calf muscles in elderly people and patients with peripheral arterial occlusive disease after ischemia reperfusion experiments [24-26]. Further, we could measure an effect of ischemic preconditioning in healthy subjects on BOLD fMRI peak signal during reperfusion [27].

## Methods

A two period placebo controlled, observer blinded, randomized crossover trial was performed in 12 healthy male subjects between 18 and 46 years (age [mean ± standard deviation] 28±6 years, body mass index 22.6±2.1 kg/m^2^). Functional MRI signals could not be analysed in one participant for technical reasons. Therefore, data are shown for 11 subjects. The study protocol was approved by the Ethics Committee of the Medical University of Vienna. All participants gave written informed consent prior to inclusion. One screening visit and two study periods with a washout time of at least 10 days in-between were scheduled for each participant. Physical examination and medical history, routine blood works including blood count, clinical chemistry, urine analysis and a 12-lead electrocardiogram were performed at the screening visit one week prior to the first study day. Subjects did not take any medication throughout the study and abstained from alcohol, stimulating beverages containing xanthine derivatives (tea, coffee) and heavy physical exercise 48 hours before drug infusion and fMRI. Subjects were fasted on study days. After the last treatment a final follow-up examination was performed.

A single dose of 1 mg/kg heme arginate or placebo (sodium chloride) was administered as an intravenous infusion in the alternate study periods. Heme arginate was diluted to 110 ml with 0.9% NaCl. The infusion was administered within 15 minutes at an infusion rate of 440 ml/h using an infusomat (IP 85–2, Döring, Munich, Germany). A post-treatment infusion of 250 ml 0.9% NaCl was administered to rinse perfusion lines.

Each study period consisted of three days. On the first day, placebo or heme arginate were administered. On the second day, the fMRI and ischemia reperfusion protocol was performed. On the third day, a blood draw for analysis of creatine kinase, lactate dehydrogenase, total protein, blood iron and C – reactive proteine as well as a physical examination were done.

Ischemia was administered to the right lower leg for 20 minutes using a cuff inflated to 200 mmHg on the thigh (1b). The cuff was deflated to 30 mmHg below systolic pressure for five minutes for slow reperfusion, mimicking impaired blood perfusion through the femoral artery. This setting has been validated previously [27]. A blood draw for NO_2_/NO_3_ analysis was performed before heme arginate infusion as well as prior to and 15 minutes after ischemia.

**Figure 1:**
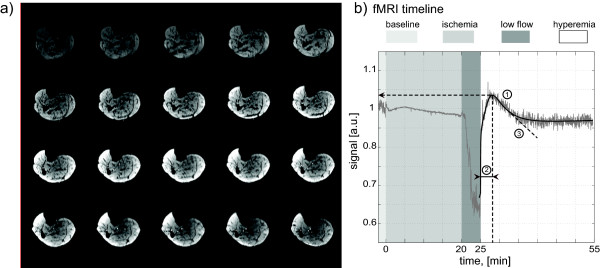
**Experimental setup.a**) EPI images for every 4^th^ minute during the experiment; **b**) fMRI timeline, a sample data set and description of parameters derived from fitted curves. Caption: Time course is showed in minutes; 1: peak BOLD signal; 2: time to peak; 3: tangent slope; a.u.: arbitrary units; min: minutes.

MR operators were blinded to the randomization sequence. A Siemens Tim Trio (Siemens Medical Solutions, Erlangen, Germany) was used for scanning. Anatomical images were acquired at the beginning of the MR examination. BOLD-imaging (EPI) had the following parameters: 128 x 128 matrix, twenty 5 mm axial slices, 1,42 mm in-plane resolution, T_R_ = 2 s, T_E_ = 28 ms. 1700 EPI stacks were acquired every T_R_. Imaging started two minutes before ischemia and continued for at least 25 minutes after reperfusion (1a + b). Regions of interest (ROI) were drawn around the soleus, gastrocnemius and tibialis anterior muscle for signal analysis. The BOLD signals in the ROIs as well as from all voxels were summed to achieve a single time course for each individual muscle and the complete calf respectively. The starting point of reperfusion was determined manually in all individual data sets.

To describe the BOLD response in calf muscle during reperfusion, the intensity time courses were fitted against a similar function as is used in DCE first pass perfusion measurements using Matlab (Mathworks, Natick, MA, USA) using the Curve Fitting Toolbox (Schewzow et al., submitted):

$f\left(t\right)=g\left(t\right)+s\left(t\right)+l\left(t\right)$

is the sum of gamma variate function *g*(*t*)

$g\left(t\right)=g_{0}\cdot {\left(t-t_{0}\right)}^{g_{l}}\cdot {\text{e}}^{-g_{2}\left(t-t_{0}\right)}$

and a sigmoid function *s(t)*

$s\left(t\right)=s_{0}\cdot \frac{1}{1+{{\text{e}}^{-s_{I}}}^{\left(t-t_{0}-t_{I}\right)}}$

A linear function *l(t)* was also added to take into account a possible drift of the signal during the experiment

$l\left(t\right)=l_{0}+l_{l}\left(t-t_{0}\right)\;$

The values of peak BOLD signal, time to peak and tangent slope were determined numerically from the fitted curves f(t) (1b).

The effect of heme arginate infusion on nitric oxide synthase was measured by NO_2_ and NO_3_ plasma concentration prior to heme arginate administration as well as prior to and 15 minutes after ischemia as previously described [28].

Descriptive statistics were used for outcome and safety parameters. To detect a treatment effect in a continuous outcome parameter of one standard deviation by a paired *t*-test with a two-sided significance level of 5%, 12 subjects had to be recruited to achieve a power of approximately 90%. With a maximum of two subjects dropping out (16.7%), power is still at 80%. The fitted measures time to peak, peak BOLD signal and slope of reperfusion signal decline of each muscle of interest were analyzed as dependent variables using a general linear mixed model, including the fixed factors ‘treatment day’ (placebo or administration of heme arginate) and muscle (soleus, gastrocnemius and tibialis anterior), and the random factor ‘subject’. An interaction of treatment day and muscle was evaluated and dropped from the model if not significant. Pairwise post-hoc comparisons between muscles were corrected for multiple testing using the Fisher’s least significant difference procedure. A two-sided p-value lower than 0.05 was considered indicating statistical significance. PASW 18.0 (SPSS Inc., Chicago, Illinois, USA) was used for statistical computations.

## Results

HA was tolerated well without adverse reaction. No significant difference of vital signs or safety blood parameters could be detected between study periods (Table 1). An increased creatine kinase serum concentration of up to 490 U/l occurred 24 hours after ischemia in two different subjects (one receiving HA, one receiving placebo) but resolved spontaneously.

**Table 1:** Vital signs in treatment periods with placebo or heme arginate

	**Placebo**	**Heme arginate**
Systolic blood pressure pre ischemia (mmHg)	115±10	114±8
Diastolic blood pressure pre ischemia (mmHg)	80±8	77±8
Heart rate pre ischemia (bpm)	73±4	72±4
Systolic blood pressure post ischemia (mmHg)	116±10	115±9
Diastolic blood pressure post ischemia (mmHg)	81±7	80±10
Heart rate post ischemia (bpm)	71±2	70±3
Blood iron (μg/dl)	110±41	100±38
Protein (g/l)	76.0±3.8	75.9±2.3
Lactate dehydrogenase (U/l)	152±28	154±28
Creatine kinase (U/l)	148±114	146±87
C – reactive protein (mg/dl)	0.06±0.05	0.08±0.09

Data are presented as mean±SD, n = 12.

Heme arginate administration 24 hours prior to ischemia increased peak BOLD reperfusion signal from 104.5±0.6% to 106.2±0.6% compared to placebo (2d; p = 0.025). Furthermore, the time to peak reperfusion was reduced from 221±19 seconds to 175±16 seconds versus placebo (p = 0.012). The calculated slope of reperfusion signal decline after peak was significantly steeper after heme arginate compared to control condition (−2.8*10^-4^±0.2*10^-4^ vs. -2.1*10^-4^±0.2*10^-4^; p = 0.002). There was no significant effect of heme arginate on the BOLD signal during ischemia.

**Figure 2:**
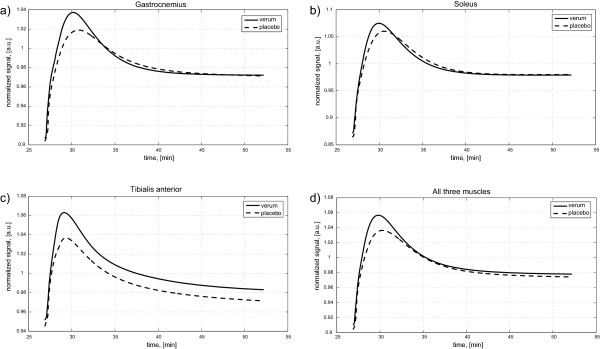
**BOLD signal during reperfusion – group average showing treatment effect.a**) gastrocnemius muscle; **b**) soleus muscle; **c**) tibialis anterior muscle; **d**) all three muscles. Caption: Reperfusion signal was fitted according to the described protocol; a.u.: arbitrary units, normalized to the pre-ischemic signal; min: minutes.

Reperfusion parameters for single muscle groups are depicted in 2a-c and summarized in Table 2. Peak reperfusion signal after placebo was highest in the soleus muscle and lowest in the gastrocnemius muscle (106.5±0.7% vs. 102.6±0.8%, p = 0.019). A similar reaction across calf muscles to heme arginate could be confirmed by statistical analysis.

**Table 2:** BOLD signal of calf muscles after ischemia-reperfusion injury

**All muscles**	**TTP (s)**	**PEAK (%)**	**slope (1/s)**
placebo	221±19	104.5±0.6	−2.1*10^-4^±0.2*10^-4^
HA	175±16	106.2±0.6	−2.8*10^-4^±0.2*10^-4^
p-value	0.012	0.025	0.002
**gastrocnemius**			
placebo	250±36	102.6±0.8	−1.6*10^-4^±0.2*10^-4^
HA	198±11	104.0±0.6	−2.2*10^-4^±0.2*10^-4^
**soleus**			
Placebo	244±31	106.5±0.7	−2.6*10^-4^±0.4*10^-4^
HA	182±8	107.8±0.8	−3.2*10^-4^±0.4*10^-4^
**Tibialis ant.**			
Placebo	169±28	104.3±1.2	−2.0*10^-4^±0.3*10^-4^
HA	152±13	106.9±1.5	−3.0*10^-4^±0.4*10^-4^

Heme arginate or placebo were administered 24 hours prior to ischemia. Data are mean±SD, n = 11; p-value was calculated using a general linear mixed model and applies to all comparisons, since interaction analysis suggested homogeneity of the treatment effect across muscles. *TTP*: time to peak; *PEAK*: peak reperfusion signal; slope: slope of the tangent at the inflection point of the BOLD signal curve after the peak; *HA*: heme arginate.

Plasma concentrations of NO_2_ and NO_3_ were 2.7±1.4 μmol/l and 13.1±8.3 μmol/l 24 hours after heme arginate administration compared to 3.5±1.8 μmol/l and 12.4±8.3 μmol/l after placebo, respectively (p = not significant). Plasma concentrations of NO_2_ and NO_3_ before heme arginate administration and after ischemia were also unchanged.

## Discussion

This study demonstrates beneficial effects of HA pre-treatment on endothelial function and reperfusion patterns after IRI in healthy humans. Full reperfusion signal occurred earlier, was higher and declined faster compared to placebo. We have previously reported that BOLD fMRI is able to characterize reperfusion patterns and the protective effects of ischemic preconditioning in IRI experiments [27]. Furthermore, we have shown the ability of HA to induce heme oxygenase-1 mRNA and protein in humans [21]. The induction of HO-1 proved beneficial in several ischemia reperfusion models in animal studies [11,17,19]. However, the application of HA against IRI in humans has not been reported yet.

We propose a stronger and earlier peak BOLD signal during reperfusion as the healthy response. This hypothesis is supported by two prior trials showing a decreased BOLD fMRI signal in elderly subjects [25,26]. Furthermore, subjects with overt peripheral artery occlusive disease presented with decreased and delayed peak BOLD signal during reperfusion [24]. HA increased the peak reperfusion signal by 38% compared to the increase over baseline after placebo administration. This is in line with one report showing a 44% higher peak signal in healthy subjects compared to the BOLD signal increase during reperfusion in elderly subjects [26]. We could furthermore demonstrate a faster decline of the reactive hyperemia signal after the peak in the HA period, indicated by a steeper decline of the slope in the infliction point of the reperfusion signal curve.

The BOLD fMRI signal is determined by a combined influence of perfusion, vascular microarchitecture and oxygen saturation of hemoglobin and myoglobin. Unfortunately, the individual contributions of each of these factors to the finally observed signal cannot be separated. Regions of interest were drawn around muscles to exclude confounding signal influences from main vessels, fatty tissue and bones. The soleus muscle, a slow-twitch fibre muscle, is known to show a high capillary density [29]. It showed the highest peak reperfusion value, as also reported in previous trials [26,27]. Vasodilation of muscular capillaries may therefore be a strong contributor to the BOLD signal. Since this trial had a crossover design and results were analysed with a repeated measurement model, an influence of vessel architecture to the different peak signals is unlikely. Improved perfusion may therefore be the major contributor to the peak BOLD signal. However, muscular metabolism may also influence oxygen extraction and hemoglobin saturation during reperfusion [27]. Therefore, the BOLD signal cannot be directly compared to reperfusion patterns measured by flow-mediated dilation or strain gauge plethysmography [30]. It seems plausible that a protected endothelial cell function is responsible for faster and increased maximal reperfusion, leading also to more rapid and increased oxygen saturation in the muscles under study. The faster normalization of the reperfusion signal after peak may be an additional sign of protected endothelial cell function and adequate early reperfusion of muscular tissue. This protection may be mediated by decreased heme toxicity and increased antioxidative capacity due to HO-1 induction and the action of its enzymatic products biliverdin/bilirubin and carbon monoxide [11]. Ischemia leads to a stasis of red blood cells in the ischemic tissue. Red blood cells are subjective to reactive oxygen radicals which are increasingly produced during ischemia and early reperfusion [31,32].

A limitation of this trial is the short period of ischemia. An increased ischemic period may reveal greater tissue injury and alter the protective effects of HA administration but is not feasible due to ethical concerns. Further, we have not measured HO-1 mRNA and protein levels in this trial. Clinical studies in patients with acute ischemic events are mandatory to validate these experiments accordingly. To draw more general conclusions, a female group and subjects with different underlying diseases should be included in future studies.

Arginine, the substrate of nitric oxide synthase, may exert systemic vasodilator effects mediated by nitric oxide [33]. To analyse a possible contribution of increased nitric oxide synthase activity induced by heme arginate, NO_2_ and NO_3_ levels were measured in plasma. The lack of changes in systemic nitric oxide metabolites argues against major vascular nitric oxide stimulation as a confounder of our results.

Our interpretation has to be discussed with caution in the context of previously published results. We reported an increased phosphocreatine overshoot and a decreased peak BOLD signal after ischemic preconditioning four hours prior to 20 minutes of ischemia. The phosphocreatine overshoot indicated an improved mitochondrial function by ischemic preconditioning, which may explain the increased oxygen demand leading to a decreased peak BOLD signal after ischemic preconditioning. Therefore, we suggest that the protecting effects of HA are different from the effects of ischemic preconditioning. The induction of HO-1 seems to be downstream in the cascade of ischemic preconditioning [34,35]. Therefore, different patterns in fMRI time series of these two protecting interventions as demonstrated by fMRI so far are reasonable. Furthermore, the fMRI measurement parameters were not identical in all studies. We increased the number of slices and decreased the slice gap for reduced artefacts from through-plane motion at the cost of reduced temporal resolution and shorter echo time. This enabled better signal-to-noise ratio but showed a slightly different contrast. Therefore, a direct, numerical comparison with our previous study is not possible [27].

## Conclusions

In conclusion, application of heme arginate improves reperfusion patterns during IRI and may protect endothelial cells and skeletal muscle against IRI in humans. The potential clinical applications of this intervention have to be addressed in further trials.

## Competing interests

The authors declare that they have no competing interests.

## Authors' contributions

All authors fulfill the criteria for authorship. MA, DD and MW designed and drafted the interventional protocol, AIS, KS and EM developed and applied the fMRI protocols. MA and SW performed ischemia reperfusion experiments. MB and GH performed laboratory analysis and supported the calculation and interpretation of endpoint measures. MA and MW wrote the final draft. All authors have read and approved submission of the final draft.
